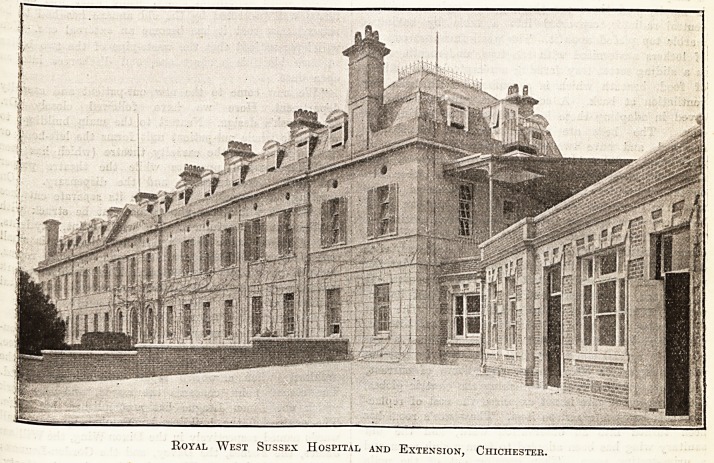# The King at Royal West Sussex Hospital

**Published:** 1913-08-09

**Authors:** 


### THE KING AT ROYAL WEST SUSSEX HOSPITAL.

### The Reconstruction Scheme Described in Detail.

The visit of the King to the Royal West Sussex Hospital, Chichester, coincides with the completion of the reconstruction scheme which has been carried out by the hospital's architect, Mr. C. W. Ball, in consultation with Dr. D. J. Mackintosh, M.V.O., superintendent of the Western Infirmary, Glasgow. That our readers may be able to see what the King saw last Saturday, and to hear the details of the interesting changes, our Commissioner visited the institution and learnt from Mr. Tom B. Harrison, the secretary, the genesis and effects of the scheme.

"The year 1910," Mr. Harrison began, "was the year in which the need for change in the institution began to be recognised, though the decision to bring the operating theatre up to date was not arrived at till 1911. Everything has worked in favour of the institution's future. The late Mr. William James gave £10,000, and it was decided to put a comprehensive reconstruction scheme in hand. The most important feature of this, from the point of view of the modern hospital, was the knocking down of the then existing sanitary blocks, and the building of two new ones; the building also of a new theatre block; and, finally, of a new out-patient unit, to say nothing of the three important balconies which have been added to the wards. As regards the cost of these reconstructions, the hospital has long had to fight with an incubus of debt, which has only been lifted in the last two years, and so it was decided not to spend a penny more than the £16,000 which was in hand, thanks to the late Mr. James's gift and to appeals which have been issued. That also explains why the reconstruction scheme leaves the number of beds stationary, although a hospital, as well as patients, gains on having one hundred beds to its credit. We are content with our capacity for seventy, and, as you will see, there were many vital matters to be done before we could tackle the question of increased accommodation. I said just now that the rebuilding of the sanitary blocks was really the most important part of the reconstruction scheme. But when I remember the necessity for replacing the old, indeed, ancient, brick floors of the basement, which were in holes and patches, and full of the accumulations of years, this need for reflooring seems the most important of all. It has been done, and the basement floor has been taken up, cleaned, and concreted, with a finish of granolithic. The plague of rats from which the hospital has suffered is now stayed, and the accommodation for the maids, which was the one part of the hospital which, for example, we should have hesitated to present for the King's inspection, is now something to be comparatively proud of. These basement rooms were always light, as the ground sloped up from them so gently; they are now hygienic and inviting. By a re-arrangement of storerooms and the removal of partition walls, we have now a scullery as large as the main kitchen, a maids' recreation rooms, and a firm concrete floor everywhere. This part of the description must not be concluded without the remark that £600 has been spent on the drainage. Let us now take the reconstructed wards and sanitary blocks in turn, and finish up with the new out-patient unit."

Royal West Sussex Hospital and Extension, Chichester.

As Mr. Harrison led the way into the Dixon Wing, as the western end of the main building is called, I inquired to what extent the ordinary work of the hospital had been interfered with.

"The rebuilding began in May 1912, for you must remember there have been extensive internal alterations also, and since then the beds in occupation have been cut down gradually to twenty-five. It was decided always to keep this number available for emergency or serious cases, and it has been a trying time both for staff and patients, especially in a sense for the former, who have had a constant experience of builders in the institution for eighteen months past."

As we walked down the passage to the Dixon Wing, Mr. Harrison pointed out the hand-lift, which serves the first floor, and the Durolite red floors, which, he said, had generally worn well and were not so cold to the feet as terrazzo. "The large ward in the Dixon Wing, like that at the other end of the building, is an L-shaped ward designed for fourteen beds. The floor is maple, and there are three open fireplaces, with a central radiator, converted into a table by having a marble top placed upon it. The ward furniture consists of lockers modernised with tile tops, underneath which is a sliding metal tray forming a receptacle for articles of food, beneath which is a cupboard with holes for ventilation at back. A considerable expense has been saved in adapting these lockers instead of buying new ones. The beds are those which Dr. Mackintosh designed, and have two wheel castors on the legs at their head, solid rubber rests on the front pair, with a rubber wheel between them, which can either rest on the ground, so that the bed can be wheeled as the two front legs are raised automatically, or can itself be raised when it is required to keep the bed stationary. They are manufactured by Nesbit, Evans and Co. The other furniture consists of a large ward cupboard, with doors all round, so that every part of the interior is easily reached, with a tile top for table purposes. It is, perhaps, a question whether this would not be improved if it too were fitted with castors. The rest of the wing consists of a linen room and ward kitchen, which has been slightly enlarged and contains a gas ring on a special stand, and also an electric heater, but while this last is cheap in its expenditure of current, there is a practical risk of breaking the electric globes, and where great care is not exercised the cost of replacing them becomes a serious item. The sister's room has been turned into an observation ward, and the old sanitary wing has been adapted to form a sink room and a urine-testing room. Then opening out of the main ward is one of the fine new balconies, sufficiently large, as you see, to take six beds. These balconies have been provided by the personal friends of the late Mr. William James as a memorial to him. The Nightingale Wing at the other or eastern end of the building corresponds to this. You will observe that the design of the hospital originally was a long two-storeyed building, with wards along the front opening out of a straight corridor that traversed it parallel to the outside walls. On the back side of this corridor opened the theatre and the sanitary wings. It is these which we have now to go over."

Beside the lift which comes up to the first floor are two smooth surface teak doors—the doors of the theatre. "The theatre unit," Mr. Harrison explains, "consists of a central hall, round which are grouped a surgeon's robing room, an anæsthetic room, the theatre itself, and the sterilising room, which was the theatre itself originally. Opalite tiles cover walls and ceiling throughout, and the heating of the theatre is given by a series of copper pipes, which form radiators. The windows are of the double-catch pattern. The theatre has been furnished by Mrs. James in memory of her husband."

"What is the main feature of the sanitary blocks?"

"They have been built out so as to allow cross ventilation between them and the main building. There is the passage leading to them with windows on each side, then in the ante-chamber two basins on one side and the door into the bath-room on the other, while in front are a sink room and two water-closets, and in one case an external projecting cupboard for bed-pans also opening out of the bath-room, and, with no other means of access, is a third water-closet, for the use of the senior staff. Overhead is a loft. The floors in the annexes are of terrazzo. These blocks have been, as I say, set back, away from the main building, and consequently occupy more superficial space, while what was an internal wall protected by the old annexe has had to be refaced now that it has become an external one. You will have noticed that the waste-pipe of the two basins in each block is a short one, and discharges into an open duct.

"We now come to the new out-patient and casualty department. Here we have followed closely Dr. Mackintosh's design. Nearest to the main building, to which the whole out-patient unit forms the left-hand or eastern wing, are the casualty theatre (which has been used for general purposes while the theatre proper was under reconstruction) and the dispensary. On entering the out-patient hall, with its separate entrance and exit, I think that you cannot but be struck with its light and cheerfulness. The white paint, skylights, and windows secure this, and the whole unit is small and compact, with its consulting and dressing rooms, and the $x$-ray room opening out. There is also a dark room for eye work, and a throat, nose, ear, and dental departments.

"What other points call for notice?"

"I must confine myself to two. The work of reconstruction has been supervised by a building committee, which has met once a week, in consultation with the architect, when a work's report has been presented. The second point concerns the wards, and the work which our Linen League has accomplished for them. The centre of the building is occupied by the small wards named respectively in the Dixon Wing, the William James, the Forbes, the Davey, and the Gordon-Lennox; and in the Nightingale Wing, the Alexandra, the Henty, the Evelyn James, and the Wyndham. To the Ladies' Linen League, which was formed not long ago, we owe all the new mattresses and several thousand articles. It has been a very great success, and an immense amount of enthusiasm has been created, and much extremely useful work performed. The League has done much to consolidate the interest of the neighbourhood, and once again indicated the usefulness of this branch of hospital work. Finally, now that the hospital has been brought up to date structurally, and that it has strengthened its financial position, and received a visit from the King, who was immensely pleased with all he saw, and remarked that apparently not a penny of the money spent had been wasted. We were all delighted to have our highest hopes realised by His Majesty graciously conferring upon it the title of "Royal," so that in future it is to be known as the "Royal West Sussex Hospital, Chichester."

## Figures and Tables

**Figure f1:**